# Dental Material Selection for the Additive Manufacturing of Removable Complete Dentures (RCD)

**DOI:** 10.3390/ijms24076432

**Published:** 2023-03-29

**Authors:** Dmitry I. Grachev, Evgeny A. Chizhmakov, Dmitry Yu. Stepanov, Dmitry G. Buslovich, Ibragim V. Khulaev, Aslan V. Deshev, Levon G. Kirakosyan, Anatoly S. Arutyunov, Svetlana Yu. Kardanova, Konstantin S. Panin, Sergey V. Panin

**Affiliations:** 1 Digital Dentistry Department, A.I. Yevdokimov Moscow State University of Medicine and Dentistry, 127473 Moscow, Russia; 2 Prosthodontics Technology Department, A.I. Yevdokimov Moscow State University of Medicine and Dentistry, 127473 Moscow, Russia; 3 Laboratory of Mechanics of Polymer Composite Materials, Institute of Strength Physics and Materials Science of Siberian Branch of Russian Academy of Sciences, 634055 Tomsk, Russia; 4 Laboratory of Nanobioengineering, Institute of Strength Physics and Materials Science of Siberian Branch of Russian Academy of Sciences, 634055 Tomsk, Russia; 5 Institute of Dentistry and Maxillofacial Surgery, Kabardino-Balkarian State University Named after H.M. Berbekov, 360004 Nalchik, Russia; 6 Laboratory of Digital Dentistry, Kabardino-Balkarian State University Named after H.M. Berbekov, 360004 Nalchik, Russia; 7 Department of Chemical Physics, Institute for Laser and Plasma Technologies, National Research Nuclear University MEPhI, 115409 Moscow, Russia

**Keywords:** PMMA, additive manufacturing (AM), material selection, analytic hierarchy process (AHP), VIKOR, multicriteria decision-making (MCDM), material index, ranking

## Abstract

This research addresses the development of a formalized approach to dental material selection (DMS) in manufacturing removable complete dentures (RDC). Three types of commercially available polymethyl methacrylate (PMMA) grades, processed by an identical Digital Light Processing (DLP) 3D printer, were compared. In this way, a combination of mechanical, tribological, technological, microbiological, and economic factors was assessed. The material indices were calculated to compare dental materials for a set of functional parameters related to feedstock cost. However, this did not solve the problem of simultaneous consideration of all the material indices, including their significance. The developed DMS procedure employs the extended VIKOR method, based on the analysis of interval quantitative estimations, which allowed the carrying out of a fully fledged analysis of alternatives. The proposed approach has the potential to enhance the efficiency of prosthetic treatment by optimizing the DMS procedure, taking into consideration the prosthesis design and its production route.

## 1. Introduction

Material selection is a relevant issue that is solved in various branches of science and technology, inter alia dental treatment (for example, manufacturing RCD). In this case, components are calculated according to the strength criterion (by the finite element method [1], as an example) and assigned margin factors. Therefore, reference data (primarily, manufacturers’ data sheets) should be taken into account. Typically, the most appropriate materials have to be selected for various functional applications. For this purpose, (i) the elastic modulus is considered to ensure a required stiffness level, (ii) crack resistance is controlled by fracture toughness, and (iii) corrosion resistance can be characterized either qualitatively or quantitatively according to the parameters measured by strictly regulated industrial standards, etc. In addition to physical and mechanical characteristics, designers consider (i) the material manufacturability (including the possibility of 3D printing, CAD milling, etc.), (ii) the variation of properties under heat treatment (for example, annealing)/post-build processing (additional polymerization, as an example), and (iii) machinability with various types of tools (grinding), etc. [2].

DMS with such a formalized approach can be solved if several required (target) characteristics are considered, among them: (a) physical and mechanical; (b) biological; (c) functional (color, polishability, roughness, etc.); (d) technological (processing methods, machinability, warpage); (e) cost, etc. Nevertheless, medical treatment tactics for the use of (temporal) dental prosthetics are a multifactorial problem. Therefore, the mission of DMS, including prosthesis manufacturing methods, becomes more complex. In practice, it is greatly affected by the mostly subjective relationships in the group “dentist–dental technician–patient” [3].

Despite the necessity of using temporary dentures, the attitude of maxillo-facial surgeons, dentists and patients to such structures is rather dismissive. To this end, breakdowns and complications are frequent due to medical and technical errors. In practical dentistry, there is a need for enduring polymer prostheses in the treatment of complex dental pathologies that requires an accurate and long-term examination. In particular, this is relevant for diagnosing gnathic problems, especially in cases of muscular–articular dysfunction. Therefore, such therapeutic and prophylactic orthopedic constructions play an important role in first-stage rehabilitation measures. These include periods of (i) temporary filling of a defect in the dentition, (ii) programming a new occlusion, (iii) osseointegration of dental implants, etc. [4,5,6]. Hence, the practice of using temporary dentures remains very important.

### The State of the Art in the Additive Manufacturing of RDC

RCDs for edentulous patients are typically made of PMMA [7]. However, a high concentration of free monomer (methyl methacrylate) and the possible development of allergic stomatitis is a significant drawback of this material [8].

Up-to-date CAD/CAE/CAM systems are frequently implemented into dental practice. They provide (i) great shape and dimensional accuracy, (ii) reproducibility, (iii) minimization of medical and technical routine work, (iv) cost reduction, and therefore (v) the availability of highly efficient dentures and facial epithesis [9,10]. However, additive manufacturing (AM) demands novel classes of polymers. In addition, the CAM process (as a subtractive method) possesses the following drawbacks: (i) material waste due to grinding and milling, (ii) wear of cutting tools and expensive equipment, (iii) restrictions on the sizes of the blanks (in the form of blocks) which does not allow the fabrication, for example, of volumetric jaw prostheses-obturators [11].

In this way, 3D printing is an actual trend in dental practice. Studies of physical–mechanical characteristics and aesthetics of such volumetric structures are highly controversial when polymeric prostheses or medical devices must be designed for long-term use (for example, in the case of parafunctional phenomena, or when an occlusion is to be reprogrammed) [12]. The quality of the AM structures depends on 3D printing parameters, building accuracy, shape, and dimensions of a virtual model, among many others [13]. Currently, a wide variety of 3D printers are available, including various physical principles of layer-by-layer deposition. To this end, prosthesis accuracy is a tangible drawback [14,15]. However, the prospects of AM in dentistry are beyond dispute [16].

Laser stereolithography apparatus (SLA) employs liquid photopolymers that are cured with a laser beam or an ultraviolet (UV) source of a certain wavelength. Product quality is affected by the dimensions of a prototype, the angle of the 3D-printed object related to the platform, the locations of supports, etc. Besides economic efficiency, such procedures are convenient for planning surgical operations with parts of a complex shape and structure [17].

The durability of (temporary) RDC depends on their design features, physical–chemical nature of the structural materials, and production routes [18]. Karaokutan et al. studied the influence of manufacturing techniques for provisional PMMA-based crowns on their strength characteristics [19]. The authors reported that computer-controlled milling improves the strength of the temporary RDC, compared to those fabricated by a direct manufacturing method. Alt et al. presented a comparative study of the strength characteristics of temporary polymer bridges made by conventional and digital technologies and concluded that the manufacturing methods substantially affect their values [20].

Dikova showed that high dimensional accuracy and surface smoothness of fixed dentures can be achieved when the vertical axis of teeth coincides with the *Z* axis of a platform [21]. At the same time, the number of supports should be increased (at least four per tooth) to reduce warpage in 3D printing and post-build polymerization. Thus, to ensure a high-quality product in designing and planning the process, it is important to consider the following: (i) printer characteristics, (ii) model placement, (iii) number of supports, and (iv) dimensional variation during and after polymerization.

Li suggested that the high-quality manufacture of temporary polymer prostheses be provided by the SLA method based on temperature-controlled layer-by-layer deposition in 3D printing (TCMIP-SL) [22]. The TCMIP-SL process contributes to the deposition of high-viscosity polymers with excellent accuracy at high speeds. 

Based on the above, it can be stated that searching for dental AM materials with improved quality has moved into the phase of developing optimal dental technologies that use industrial polymers and help to minimize fabrication process disruptions that deteriorate a product’s characteristics.

This paper addresses the development of a formalized approach for DMS and the additive manufacturing of RCD. This issue was solved using a decision-making methodology. Rational ranking was illustrated on examples of three types of commercially available PMMA grades processed by the identical DLP method. The paper is structured as follows. Section 2 describes the 3D printing method and techniques for evaluating the key properties of the AM blanks. Section 3 contains the measurement results for various characteristics of dental materials; the calculation of the material indices is also provided. Section 4 proposes the approach to multicriteria optimization in DMS and examples of their ranking as well. Section 5 discusses the obtained data. The authors proposed, based on examples of certain industrially produced brands, an approach to (or the tool for) brand ranking; the variability of the results was emphasized. Recommendations to use one or another brand of dental materials remain for individual consideration. 

## 2. Materials and Methods

### 2.1. Three-dimensional Printing (Materials and Equipment)

The test samples were fabricated in two stages: modeling and 3D printing. Virtual master models were created using the ExoCad Gateway 3.0 software (Align Technology, San Jose, CA, USA). The final files of the completed sample models (in the “*.STL” format) were imported into the (slicer) software package for the 3D printing preparation. Then, (i) the models were positioned relative to the plane of the 3D printer platform, (ii) the supports were placed, (iii) the models were divided into layers, and (iv) the 3D printing parameters were adjusted in line with recommendations of the feedstock manufacturers (Table 1).

### 2.2. Property Evaluation Methods

Comparative studies of the mechanical, tribological, technological, biological, and economic characteristics of dental materials were performed. Mechanical tests were carried out for tension, compression, and three-point bending. In this paper, only the three-point bending data are reported, since they are more informative for the dental materials [23]. The key parameters to be evaluated are flexural strength, flexural modulus, and flexural strain.

#### 2.2.1. Three-Point Bending Tests

The samples took the form of rectangular plates with dimensions of 25 × 2 × 2 mm following the Russian State Standard GOST 31574-2012. An “Instron 5982” electromechanical testing machine (Illinois Tool Works Inc., Glenview, IL, USA) was used with a crosshead speed of 0.75 mm/min. The force gauge had an upper measurement limit of ± 5000 N (series 2580–108). The span was 20 mm. Before testing, the samples were conditioned in distilled water at a temperature of 37 ± 1 °C for 24 h. The tests were carried out until the sample failure.

#### 2.2.2. Impact Strength Tests

The *a_n_* Charpy impact strength of specimens without a notch was measured with a “KM-5” pendulum impact tester (“ZIP” LLC, Ivanovo, Russia). Their sizes were 80 × 10 × 4 mm according to the Russian State Standard GOST 4647-2015. There were four specimens of each material. After the tests, the average *a_n_* values were calculated.

#### 2.2.3. Biological Tests

For biological tests, the samples were in the form of disks 5 mm in diameter and 1 mm thick. The attachment points for supports were finished with the polishes of various abrasiveness in the following sequence: 9400.204.030, 9401.204.030, 9402.204.030 (Komet, Gebr. Brasseler GmbH & Co. KG, Germany). The time lag from sample fabrication to the biological tests did not exceed 72 h. Immediately before the start of the in vitro experiment, the samples were cleaned in an ultrasonic bath for 15 min, after they were treated with 70% ethanol.

To carry out the process of the primary adhesion of microorganisms, the samples were placed in test tubes with a suspension of the test strains of the corresponding species at a standard concentration. We used the optical turbidity standard of 0.5 U McFarland, which corresponded to 10^9^ colony-forming units (CFU)/mL for bacterial cultures and 10^8^ CFU/mL for yeast ones. After quantitative inoculation, bacteria were cultivated under anaerobic conditions at a temperature of 37 °C for 7 days, and fungi—at room temperature (25 °C) for 2 days. Adhesion indices were determined as a ratio of the decimal logarithm of the number of CFU obtained after sonication of the test samples to the decimal logarithm of the CFU of the initial microbial suspension. The authors described the experimental technique in detail in their previous paper [7].

#### 2.2.4. Tribological Tests

In the point tribological contact according to the “ball-on-disk” scheme, the dry sliding friction tests were carried out at a load (*P*) of 5 N and a sliding speed (*V*) of 0.3 m/s. A “CH 2000” tribometer (CSEM, Neuchâtel, Switzerland) was used following ASTM G99. The tribological tests were conducted using a ceramic counterpart (a ball made of the ZrO_2_) with a diameter of 6 mm and the *R_a_* surface roughness of 0.02 µm. The latter was assessed with the “New View 6200” optical interferential profilometer (Zygo Corporation, Middlefield, CT, USA). The testing distance was 1 km and the tribological track radius was 10 mm.

In the linear tribological contact according to the “block-on-ring” scheme, dry sliding friction tests were performed using a “2070 SMT-1” friction testing machine (Tochpribor Production Association, Ivanovo, Russia). A load (P) was 60 N, while a sliding speed (V) was 0.3 m/s. A ceramic counterpart was made of an Al_2_O_3_ ring with a diameter of 35 mm and a width of 11 mm. The *R_a_* surface roughness was 0.20 µm. The counterpart temperatures were assessed with the “CEM DT-820” non-contact infrared (IR) thermometer (Shenzhen Everbest Machinery Industry Co., Ltd., Shenzhen, China).

WR levels were determined by measuring the width and depth of wear tracks according to a stylus profilometry (KLA-Tencor, Milpitas, CA, USA), followed by multiplication by their length. They were calculated taking into account load and distance values:

(1) $$\mathrm{Wear}\;\mathrm{rate}\;=\frac{\mathrm{volume}\;\mathrm{loss}\;\left({\mathrm{mm}}^{3}\right)}{\mathrm{load}\;\left(N\right)\times \mathrm{sliding}\;\mathrm{distance}\;\left(m\right)}$$

**Figure 1:**
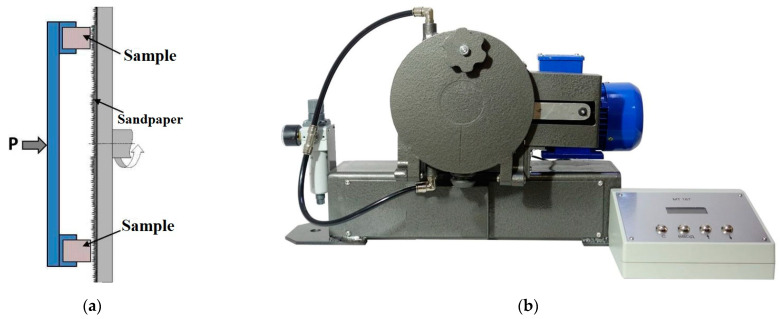
A scheme of the abrasive wear tests (**a**) and the “MI-2” abrasion testing machine (**b**).

In the flat tribological contact, abrasion wear tests were conducted. The “MI-2” abrasion testing machine (Metroteks LLC, Moscow, Russia) was used to determine the weight loss values at abrasion by fixed particles, according to the “polymer pin-on-abrasive disk” scheme (Figure 1b), regulated by the Russian State Standard GOST 426-77. The dimensions of the samples were 8 × 10 × 8 mm. The average grain size of an abrasive paper (P1000) was ~17 µm. The angular sliding velocity was 40 rpm, and the load was 10 N. The test scheme is shown in Figure 1a. 

Weight loss was determined every 5 min during the total test duration of 20 min. The samples were weighed using the “Sartogosm LV 120-A” (Sartogosm LLC, Saint Petersburg, Russia) with an accuracy of 0.1 mg. 

#### 2.2.5. Polishability (via Roughness)

The protocol for grinding and polishing in the sample preparation procedure is presented below.

1. Surface treatment with a carbide cutter for polymers until the required configuration or shape.
2. Surface treatment with a carbide cutter for polymers to remove surface irregularities.
3. Sanding with 180–220 grit sandpaper for extra fine finishing.
4. Finishing with a felt and a moistened polishing powder.
5. Brushing with a grinder using a coarse bristle and a moistened polishing powder for a smooth surface.
6. Processing with a grinder using a thread brush and a fine-grained polishing paste to a mirror finish.

The *R_a_* surface roughness was assessed with the “New View 6200” optical interferential profilometer (Zygo Corporation, Middlefield, CT, USA).

## 3. Results

### 3.1. Mechanical Properties

The mechanical properties of the SLA 3D-printed PMMA samples, registered in the three-point bending tests, are presented in Table 2. For the Dental Sand (DS), the flexural strength and strain values exceed by ~10 % and two times the corresponding characteristics for Free Point (FP), as well as by 2.5 and 3.6 times for Nolatech (NT), respectively. For all the studied PMMA grades, the flexural modulus values were at the same level of about 2.7 ± 0.1 GPa.

Since PMMA RCD could experience impact in use (for example, being accidentally dropped on ceramic tiles), their impact strength could be an important performance characteristic. The conducted Charpy impact tests showed that *a_n_* values were 0 J/cm^2^ at the minimum applied impact energies regardless of the PMMA grade. Thus, it was impossible to differentiate the materials by this parameter. Please note that an increase in impact strength could be achieved by loading PMMA with fibers or nanoparticles in various concentrations, but this would change the manufacturability of the materials, including the possibility of their AM processing [24,25,26].

### 3.2. Biological Properties

The adhesion indices (AIs) of the normal, periodontopathogenic, and fungal microbiota to the studied materials are presented in Table 3. In the normal microbiota case, no differences were found between the FP (0.55 ± 0.06) and the NT (0.56 ± 0.06). For the DS, a value of 0.43 ± 0.06 was noticeably lower than those for the other two materials. For the periodontopathogenic microbiota, an AI value of 0.42 ± 0.05 for the NT was noticeably higher than those for the DS and the FP, for which no significant differences were found (AI = 0.34 ± 0.05). In the fungal microbiota case, some variations were observed for all the materials. The maximum level was typical for the NT (AI = 0.49 ± 0.05), and the minimum was detected for the DS (AI = 0.34 ± 0.05).

### 3.3. Tribological Properties

#### 3.3.1. The Point Tribological Contact

The point tribological contact involved a local impact of the ceramic ball on the polymer sample (in the form of a disk) under dry sliding friction conditions. Table 4 presents the values of the tribological properties of the materials under study. The FP and DS had the lowest coefficient of friction (CoF) values (0.276 and 0.271, respectively), while it was higher by 10 % (0.303) for the NT (Figure S1). The wear rate (WR) levels were also evaluated. For the FP, it was half that for the NT and DS. Roughness on the wear friction track surfaces was approximately at the same level of 0.19 µm regardless of the PMMA grade.

#### 3.3.2. The Linear Tribological Contact

In the linear tribological contact, the ring-shaped ceramic counterpart slid relative to the stationary polymer samples, along the “non-renewable” surface of the wear tracks. Therefore, the specific pressure was noticeably lower compared to that in the point tribological contact. As a result, the average CoF values were lower by ~3 times for all the studied materials (0.131, 0.096, and 0.122, respectively, according to Table 5). The NT had the lowest WR level of 0.078 × 10^–6^ mm^3^/N·m, which was 2.3 times lower compared to that for the DS and 1.5 times than that for the FP (Figure S2). In contrast to the point tribological contact, the WR values were an order of magnitude lower (10^–5^ and 10^–6^, respectively).

#### 3.3.3. The Flat Tribological Contact, Abrasive Wear

Since PMMA prostheses could be worn out by hard particles, abrasive wear tests were conducted according to the “polymer pin-on-abrasive disk” scheme. It was shown that the least wear (weight loss) was observed for the FP (0.12 mg) after 20 min of testing (with abrasive particles fixed on the non-renewable surface of the abrasive counterpart), which was 1.6 times less compared to that for the DS (0.19 mg) and 2.0 times less compared to the NT (0.25 mg) (Figure S3).

Table 6 summarizes the data on the WR values in the point, linear, and flat tribological contacts used to calculate the material indices.

### 3.4. Technological Properties

A significant number of the parameters could be qualified as “technological properties”. Since the study deals with DMS concept development, to simplify the process, the authors limited themselves to only three ones (Table 7).

The first parameter was determined by the average duration of 3D printing and post-build polymerization processing. Its minimum value was typical for the FP (*t* = 63 min), while the maximum for the DS was (*t* = 110 min). 

The second parameter was polishability, which was related to the ability of the 3D-printed PMMA products to achieve the required degree of gloss, determined by the surface roughness. In general, all the studied materials could be considered to be similar in terms of their values (*R_a_* ~ 0.048–0.051 µm). 

The third parameter was shape distortion (warpage) [27]. This was assessed qualitatively, being associated with the ability of a 3D-printed product to retain its shape after cooling. From this point of view, the NT was the only material characterized by warpage after 3D printing and post-build polymerization processing. To use these data as a quantitative criterion, this parameter was assigned at a level of 0 in the absence of the shape distortion, and otherwise it was 1.

### 3.5. Economic Indicator (Cost)

The financial aspects of manufacturing PMMA prostheses via 3D printing could also be assessed by numerous criteria, including costs of logistics, purchasing licenses, deployment of a particular type of 3D printer and its maintenance, and many others. Nevertheless, the authors implemented the only criterion, namely the feedstock cost, for simplification (since the sample fabrication was carried out with the same 3D printer). Quantitative data are given in Table 8 for all the materials under study.

### 3.6. Ranking Materials by Indices

**Figure 2:**
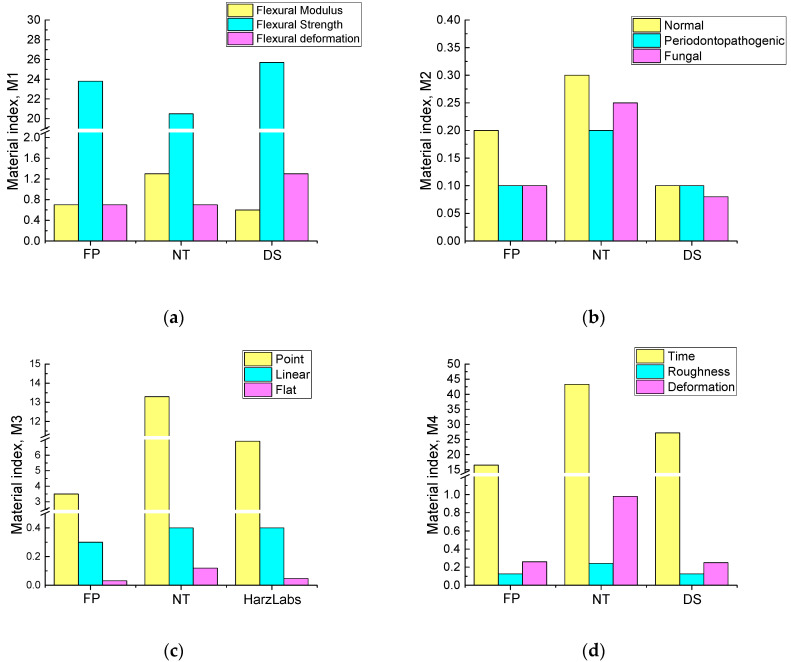
The material indices of the studied dental materials: M1 is the ratio of the mechanical properties to the feedstock cost (**a**), M2 is the ratio of the biological properties to the feedstock cost (**b**), M3 is the ratio of the tribological properties to the feedstock cost (**c**), M4 is the ratio of the technological properties to the feedstock cost (**d**).

The following parameters were introduced in the study according to the concept of the material indices proposed by Ashby [2] (as a ratio of the data presented in Table 2, Table 3, Table 4, Table 5, Table 6 and Table 7 to the feedstock costs in Table 8). The charts of the material indices are shown in Figure 2. Their values were obtained by dividing the factors by the feedstock costs and multiplying by 100, they are:

- M1 is the ratio of the mechanical properties to the feedstock cost (namely flexural modulus, flexural strength, and flexural strain);
- M2 is the ratio of the biological properties to the feedstock cost (all three types of the studied microbiota were considered);
- M3 is the ratio of the tribological properties to the feedstock cost (a wear resistance for all three schemes of the tribological tests);
- M4 is the ratio of the technological properties to the feedstock cost (the average duration of 3D printing and post-build polymerization processing, roughness after standard polishing, and warpage after 3D printing).

To this end, the use of the concept of material indices (Tables S1–S4) offered a clear tool for quantitative comparison of the dental materials in the case under study. Moreover, it was possible to choose (from the point of view of a user or an expert) a more or less significant one. However, the significance factor was very subjective, so the analysis had to be carried out either by considering the data in a multilevel space or using multicriteria optimization approaches [28].

In the first case, an efficient method could be implemented to reduce the dimension of the analyzed data space, e.g., down to two [29,30,31,32]. The solution using the second approach is introduced in the next section.

## 4. Data Interpretation—The Combined AHP–Extended VIKOR Methods

In this section, some methods for DMS were compared, taking into account their production routes, which provided a trade-off between requirements for a set of mechanical, tribological, technological, biological, and economic criteria. The authors used informal subjective assessments of experts in the field of dental prosthetics, 3D printing, and the manufacture of CRD by subtractive and additive methods (primarily at A.I. Yevdokimov Moscow State University of Medicine and Dentistry, Russia).

### 4.1. The Problem Statement and Methods

Within the decision-making theory framework, the studied dental materials were qualified as decision alternatives with their designation as *A_i_*. The factors characterizing each alternative were quantitative assessments and qualitative indicators. Based on the factors, if the criteria of (i) quality, (ii) usefulness, (iii) reliability, etc. were put forward, then the alternatives could be compared. The problem of choosing an alternative arose when there was a contradiction between the results of comparison or the absence of an alternative with the best indicators of the factors (an ideal combination of the characteristics) [33]. In this case, the problem of multicriteria optimization arose, namely the choice of a rational alternative from the available finite set, i.e., an alternative that was closest to an “ideal” option.

To date, a large number of methods for solving multicriteria optimization problems are known [34,35], i.e., Multicriteria Decision-Making (MCDM) methods. They include the Analytic Hierarchy Process (AHP), Technique for Order of Preference by Similarity to Ideal Solution (TOPSIS), VIKOR, ÉLimination Et Choix Traduisant la REalité (ELECTRE), Preference Ranking for Organization Method for Enrichment Evaluation (PROMETHEE), etc. The key difference between these methods lies in the algorithms bringing different-scale, often qualitative, data into a single normalized space and the subsequent choice of a metric inside it. Examples of MCDM can be found both in tribology [36] and medicine [37,38,39], as well as in other areas [40,41]. Recently, MCDM, based on interval estimates, has been developed. For example, extended both TOPSIS and VIKOR methods were described, [42,43] while their advantages and drawbacks were reported in [44,45,46]. In this paper, the authors consider the possibility to implement the AHP and VIKOR ones for solving the problem of the DMS (PMMA-based) for manufacturing RCD (including the temporary ones).

### 4.2. Initial Data Analysis

All the data were divided into groups according to their physical meanings (Table 9). The mechanical, tribological, technological, biological, and economic groups included the experimental data in the form of interval quantitative estimations with different scales. The remaining groups were described by point quantitative values (in contrast to the interval ones). The exception was the “Warpage after 3D printing” technological factor. It was qualitative (binary) in nature and could be coded as “0” (“no warpage”), and as “1” (“might be distorted”) in this case. Since the “Roughness after polishing” technological factor turned out to be identical for all the materials, it was not used in analysis and decision-making.

The material assessment criteria were selected separately for each factor and coded according to two principles: (+1) was the “utility” principle (“the more, the better”) and (–1) was the “cost” one (“the less, the better”).

### 4.3. Determination of Criteria Weights by the AHP Method

The AHP method was implemented to determine the weights (significance) of the criteria [47]. It referred to ones for supporting selection from a small number of alternatives based on pairwise comparisons. In this case, the formation of a matrix of the pairwise significance of the criteria was performed by an expert, and the calculation of the weights of the criteria was carried out by searching for the eigenvalues of this matrix. Due to the large number of the criteria and their different nature, the analysis of their pairwise significance was conducted within the groups, first, and between them, second. The following scale was used to assess the pairwise significance:

1—the criteria were the same;

3—the first criterion was slightly more important than the second one;

5—the first criterion was much more important than the second one;

7—the first criterion was undeniably more important than the second one, it was confirmed not only by experts but also in practice;

9—the first criterion was of absolutely greater importance than the second one.

Tables of the pairwise comparison within the groups were filled by experts from the respective fields. Therefore, despite such an assessment being subjective, the spread of opinions within the groups was low and not of interest to the research. The results of the pairwise comparison and the calculation of the weights are presented in Table 10, Table 11, Table 12 and Table 13.

### 4.4. Determination of the Criteria Weights by the VIKOR Method

To rank alternatives, the authors used the extended VIKOR method for the interval estimation [42]. The VIKOR method was based on the *L_p_* metric for normalized functions [42,46]:

(2) $$L_{pi}={\left\{\sum_{j=1}^{n}{\left[w_{j}\frac{f_{j}^{*}-f_{ij}^{}}{f_{j}^{*}-f_{j}^{-}}\right]}^{p}\right\}}^{\frac{1}{p}}$$

where $f_{ij}^{}$ is a value of the *j*-th criterion for *i*-th alternative; $f_{j}^{*}$ is the best value of *j*-th criterion among all the alternatives; $f_{j}^{-}$ is the worst value of *j*-th criterion among all the alternatives; *w_j_* is the weight of the *j*-th criterion. In calculations, two special cases of this metric (2) were applied:

(3) $$\mathrm{at}\;p=1\;S_{i}=\sum_{j=1}^{n}w_{j}\left[\frac{f_{j}^{*}-f_{ij}^{}}{f_{j}^{*}-f_{j}^{-}}\right]$$

was the weighted Manhattan distance to the ideal alternative consisting of the “best” factor values,

(4) $$\mathrm{at}\;p=\text{∞}\;R_{i}=\underset{j}{\max}w_{j}\left[\frac{f_{j}^{*}-f_{ij}^{}}{f_{j}^{*}-f_{j}^{-}}\right]$$

was the weighted Chebyshev distance.

Additionally, a weighted and normalized value was introduced as an intermediate one of the above metrics:

(5) $$Q_{i}=v\frac{S_{i}-S^{*}}{S^{-}-S^{*}}+\left(1-v\right)\frac{R_{i}-R^{*}}{R^{-}-R^{*}}$$

where $S^{*}=\underset{i}{\min}S_{i}$, $S^{-}=\underset{i}{\max}S_{i}$, $R^{*}=\underset{i}{\min}R_{i}$, $R^{-}=\underset{i}{\max}R_{i}$, *v* is the weight of the “the majority of criteria” strategy.

The *S*, *R,* and *Q* values (3–5), could be referred to as the pessimistic, optimistic, and rational assessments of the alternative position in the set, respectively. Their values were in the range of 0 to 1. For the *S*, *R,* and *Q* values, their equality to zero was the ideal combination, while the equality to 1 was the worst option.

Ranking of the alternatives was carried out by ordering $Q_{i}$ values and comparing their difference with the 1(m−1) level, where *m* was the number of alternatives.

**Figure 3:**
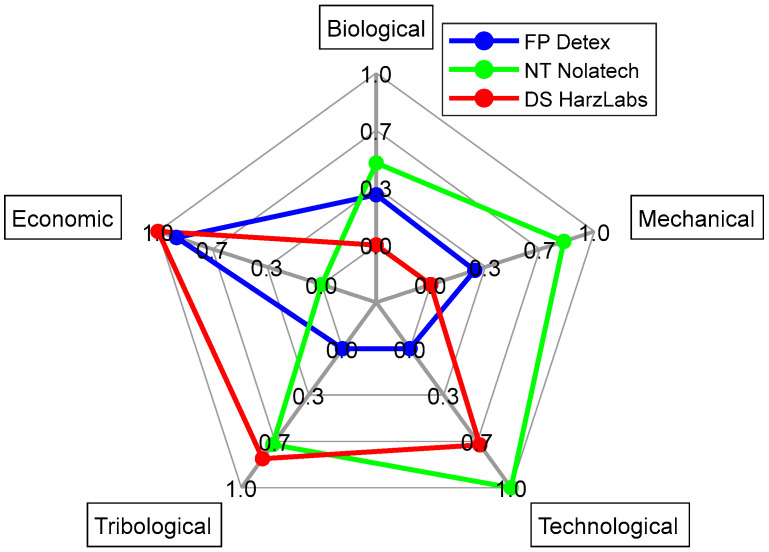
A radial diagram of *Q*_1_ calculation by the groups of the factors.

**Figure 4:**
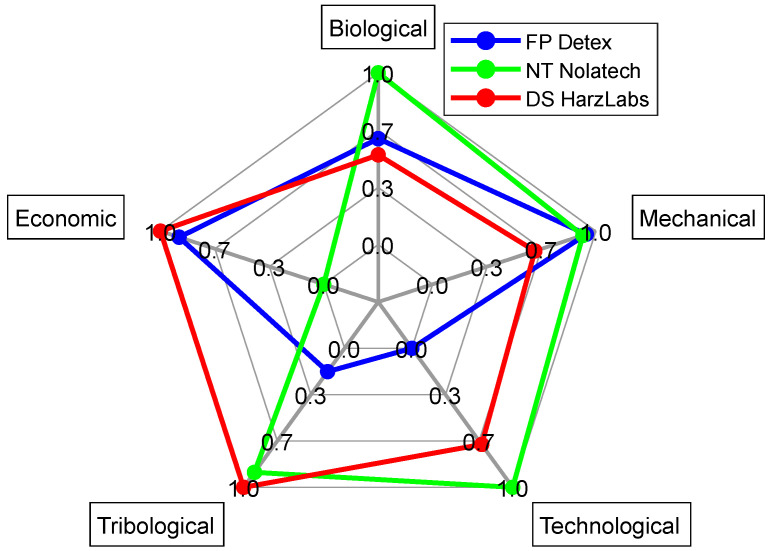
A radial diagram of *Q*_2_ calculation by the groups of the factors.

For the interval values of the factors, distances were assessed according to their boundaries [42]: $S_{i}=\left[S_{i1},S_{i2}\right]$, $R_{i}=\left[R_{i1},R_{i2}\right]$, $Q_{i}=\left[Q_{i1},Q_{i2}\right]$. The calculation results are presented in Table S5. Figure 3 and Figure 4 show the values of *Q*_1_ and *Q*_2_ at *v* = 0.5. The analysis of the lower boundary of the *Q* rational option reflected that the NT turned out to be the worst alternative, since it had the best factors only in the “economic” group out of the five ones. Both the FP and DS possessed the best factors in the two groups (Figure 3). However, there was no obvious advantage of the DS over the NT considering the *Q* top boundary (Figure 4).

### 4.5. Ranking Analysis for All Criteria

The stage of the paired comparison of the groups was the most subjective phase of the analysis. As a result, there can be achieved a coordinated decision of several experts from different subject areas at once or a single decision maker. In the latter case, the preference for the advantage of a characteristics group of a final product could be determined, for example, by the price-to-quality ratio. If the first four groups of the factors and the criteria corresponding to them characterized the product quality, then the quantitative expression of the price-to-quality indicator in the table of the pairwise comparison of the groups is proposed to express in the form of the following preference options (Table 14):

- Preference #1. The group equivalence assumption.
- Preference #2. The small advantage assumption for the “economic” group over all the others.
- Preference #3. The “economic” group was considered less significant relative to all the others.

**Table 14:** The paired comparison of the groups. Preference #1/Preference #2/Preference #3.

Group	Mechanical	Tribological	Technological	Biological	Economic
Mechanical	1/1/1	1/1/1	1/1/1	1/1/1	1/0.33/3
Tribological	1/1/1	1/1/1	1/1/1	1/1/1	1/0.33/3
Technological	1/1/1	1/1/1	1/1/1	1/1/1	1/0.33/3
Biological	1/1/1	1/1/1	1/1/1	1/1/1	1/0.33/3
Economic	1/3/0.33	1/3/0.33	1/3/0.33	1/3/0.33	1/1/1

Table 15 summarizes the results of the calculation of the weights by the AHP method of pairwise comparison for all the studied cases. As expected,

- the preference variability for the “economic” group affected the weight of the economic factor from the first rank (of importance) to the last one;
- the criteria of those factors (excluding the “economic” ones) recognized as the most significant within their groups had the highest weights. In this example, they were (i) the “periodontopathogenic” parameter from the “biological” group, (ii) the “warpage after 3D printing” from the “technological” group, and (iii) the “flexural modulus” from the “mechanical” group.

**Table 15:** Criteria weights.

Group	Factor	Preference #1		Preference #2		Preference #3	
		Weight	Order	Weight	Order	Weight	Order
Mechanical	Flexural modulus	0.085	4	0.090	4	0.074	5
	Flexural strength	0.076	6	0.081	6	0.066	7
	Flexural strain	0.076	6	0.081	6	0.066	7
Tribological	Wear rate, point contact	0.079	5	0.083	5	0.068	6
	Wear rate, linear contact	0.079	5	0.083	5	0.068	6
	(Abrasive) weight loss, flat contact	0.079	5	0.083	5	0.068	6
Technological	Average duration of 3D printing and post-build polymerization processing	0.074	7	0.078	7	0.064	8
	Warpage after 3D printing (quality)	0.091	2	0.096	2	0.078	3
Biological	Normal	0.067	8	0.071	8	0.058	9
	Periodontopathogenic	0.129	1	0.135	1	0.113	2
	Fungal	0.086	3	0.091	3	0.075	4
Economic	Price for 1 kg of feedstock	0.079	5	0.028	9	0.203	1

For all three preferences, the *S*, *R*, and *Q* values were calculated using both the VIKOR and the extended VIKOR methods [42,46]. The obtained results and the ranking data are presented in Table 16. According to the preferences:

- under the assumption of the equivalence of the groups, the extended VIKOR method did not reveal any obvious advantage of the alternatives, while the VIKOR one recognized the equal advantage of the FP and NT over the DS.
- under the assumption of the importance of the “economic” factors, the FP was recognized as a rational alternative according to the VIKOR method, but it was the NT according to the extended VIKOR one.
- under the assumption of the significance of all groups over the “economic” factors, both methods recognized the FP and DS as rational alternatives, but the NT was the worst one.

Comparing the ranks for all the preferences, it should be noted that the subjective phase of determining the significance of the criteria made a significant contribution, but the variability of the factors was no less important. As follows from Table 16, a large spread of the measured interval factors (Table 9) caused a great dispersion of *S*, *R,* and *Q* interval estimations and, accordingly, predetermined a lower “resolving capacity” of the extended VIKOR method (Table 16) at a few alternatives. Under MCDM resolving capacity, the authors meant the ability of the method to compare the alternatives and differentiate them [48].

## 5. Discussion

The photopolymerization process is well studied and widely used in industry [49,50,51,52,53,54,55,56,57]. SLA is based on the photopolymerization phenomenon as well. In particular, when the photoinitiator absorbs UV, the molecule splits into two radicals. The latter combines with monomers to form new radicals that group with other monomers. This reaction forms polymer chains to transform liquid photopolymerized resin into a solid state [58]. 

In dentistry, one of the challenges in the 3D printing of acrylate resins is residual monomers. After material curing, dental acrylates release various amounts of potentially toxic substances into saliva, where they dissolve and affect tissues of the mouth and the human body as a whole. The substances include unpolymerized, unreacted components of a chemical system, as well as secondary polymerization products. At high concentrations, they are very toxic, but their amount dissolved in the saliva is negligible when using dentures, depending on the possibility of their diffusion from the material. However, these substances may significantly affect a patient’s well-being due to individual intolerance, since acrylates are cytotoxic substances.

It is generally accepted that the residual monomers (MMA, BuMA, EMA, and UDMA) and the crosslinkers (EGDMA, IBMA, etc.), which have not fully polymerized in the material curing procedure, are responsible for the toxic and allergenic effects of the acrylates. The amount of a monomer released into the patient’s oral cavity is proportional to its total residual quantity in the matrix of the acrylates. The residual monomer diffuses rather quickly from the polyacrylate surface layer (it is released into saliva during the first day of using a denture). However, its certain amount remains “locked” inside polyacrylate for a long time, continuing to slowly diffuse outwards. The amount of the released MMA becomes stable two weeks on from the denture installation.

According to ISO 1567:1999, the maximum allowable residual MMA contents are 2.2% and 4.5% for thermal and cold-cured dental acrylates, respectively. The residual monomer amount can be reduced by post-curing (additional thermal polymerization in boiling water or a microwave oven) and extraction (immersion and holding of dentures in a water bath or sonication in water). Using microwave post-curing procedures, the residual monomer amount can be lowered by 25% (due to its polymerization and/or evaporation). According to the authors, the most promising method for its decreasing is preliminary polymerization under the action of ultraviolet or microwave radiation. In addition, new initiating systems (polymerizable monomers) should be developed.

The authors assume that the above aspect is very relevant from the standpoint of DMS and should be considered when developing such procedures. In the present research, this aspect was neglected for objective reasons. Nevertheless, it will be analyzed in a forthcoming paper by the authors, including the addition of appropriate quantitative indicators to the matrix for comparing the functional properties.

Dental materials used for the manufacture of RCD (for temporary use) must have a wide range of functional properties, which include bio-inertness; anti-allergenicity; specific color palette (including stability of shades and surface textures); physical and mechanical characteristics; good polishability; no negative reaction to hygiene products; manufacturability (simplicity and ease of processing; short duration); economic viability, etc. [59]. Some of these parameters can be characterized quantitatively, and some only qualitatively. Moreover, the achievement of the required level of some properties may be accompanied by the unattainability of those for others. Thus, the issue of DMS is very complicated and carried out empirically in most cases. This contributes to a great risk of errors, transforming into complications and aggravation of the patient’s conditions, which is confirmed by the long-term practice of doctors, reflected in the literature [60,61]. Presently, the importance of production routes is a fact that significantly affects the quality characteristics of the fabrication of dental products.

Even though most of the study was devoted to the analysis of the properties of the dental materials and their ranking based on the assessment of their characteristics, it should be noted that they were mainly determined by the structure formed during the 3D-printing process. With this method and the applied conditions for AM, the achievement of the key mechanical and tribological properties was determined by the PMMA molecular structure and the pattern of macromolecule arrangement. For this reason, the revealed difference in the properties between the three types of the studied dental materials was not associated mostly with some variations in the feedstock compositions, but with the specifics of their polymerization during the 3D printing process (taking into account high rates of the product fabrication). In addition, the important influencing factors were:

- compositions of processing additives (trade secrets of the manufacturers);
- recommended time-depended modes of 3D printing and post-build polymerization processing (differed for the studied PMMA grades);
- degrees of residual monomer contents, implemented in 3D printing and post-build polymerization processing;
- residual stresses, characterized by strains of the 3D printed samples, etc.

The mathematical algorithm implementation could contribute to the consideration of structural characteristics in the DMS. However, this would complicate the approach (tool) applied in this research, which was based on the materials’ ranking over the integral and experimentally determined characteristics (interval, quantitative or qualitative).

DMS was highly case-sensitive, depending on the preferences of an expert. Nevertheless, the proposed approach (with proper tuning) was rigorous and enabled the obtaining of quite weighted estimations. It could be effectively used for solving related problems, such as digital milling from blanks. The key aspects remained as follows:

- correct selection of the factors (groups of factors);
- ensuring the accuracy of their measurement and reducing errors (dispersions of the experimental data);
- ensuring the most representative expert assessment;
- if the risk of making a wrong decision remains informalized, the only way to minimize it is to form the right attitude of the decision maker toward expressing his/her preferences.

Note, in this research, the evaluation of the tribological performance was carried out according to the standards intended for testing structural materials (without taking into account the specifics of existing regulations for dental ones). This was not critical from the standpoint of developing an approach to DMS. However, the authors will be careful to follow the standard requirements for testing dental materials.

It should be also noted that presently the issue of DMS is solved in a very subjective way. It depends on a large number of factors: the dentist’s experience, price, patients’ budget, time availability, and particular values of a variety of functional properties. The papers proposed a concept that considers the factors formulated by experienced long-term practicing dentists. The developed approach presumes an option to make flexible corrections. In addition, it can be easily adapted for solving the DMS issue for dental implants as well. The paper illustrated it over the ranking of three commercially available PMMAs. The significance of the study concludes in attracting the mathematical tools for solving real problems of practicing dentists with a low amount of subjectivity in making a decision.

By way of summarizing, the following might be concluded. We have employed the AHP method for ranking the factors, i.e., more or less important. Furthermore, a compromise is possible to be found over the set of alternatives with the VIKOR method. The proposed approach (the tool) is of great promise to enhance the efficiency of prosthetic treatment by optimizing the DMS procedure, taking into account the prosthesis design and its production route.

## 6. Conclusions

The formalized approach to a rational ranking of dental materials aimed at RCD additive manufacturing was proposed within the framework of the multicriteria optimization algorithm. It was tested through three types of commercially available PMMAs, processed by the DLP. For this purpose, the combination of mechanical, tribological, technological, microbiological, and economic factors was assessed. The following results were obtained, and conclusions were drawn.

1. The calculation of the material indices was carried out to compare the studied dental materials for a set of functional parameters related to feedstock cost. However, this did not solve the problem of simultaneous consideration of all the material indices, inter alia their significance.
2. For the 3D printing of RCD, the problem of the DMS could be solved as a multicomponent optimization. This study solved the problem by combining the AHP and extended VIKOR methods with interval estimation.
3. It was shown that the formulated preferences by experts exerted a significant impact on the decision-making results under the conflict of the adopted criteria. The proposed method of grouping the factors according to the expert competencies allowed the reduction of subjectivity, at least at the stage of ranking within the groups. However, uncertainty arose for all criteria at the stage of alternative analysis.
4. The implementation of the extended VIKOR method, based on the analysis of interval quantitative estimations, allowed the carrying out of a fully fledged analysis of the alternatives. Its results were rather plausible. However, it was characterized by a lower “resolving capacity”, i.e., the ability to separate the alternatives.

As an outlook, the authors consider it necessary to note the following. The AHP method was employed to rank the factors over the degree of importance. Furthermore, a compromise is possible over the set of alternatives with the VIKOR method. The proposed approach is targeted at enhancing the efficiency of prosthetic treatment by optimizing the DMS procedure if the prosthesis design and its production route are taken into account. The development of the proposed approach correlates with the attraction of the MCDM, which takes the experts’ uncertainty in decision-making (estimation) into account. The methods based on fuzzy logic theory are among them [41,62,63].

## Figures and Tables

**Table 1:** The data on the 3D printing materials and methods.

Material	FreePrint, Temp 385, A2	Nolatech	Dental Sand, A1–A2
Designation in the text	FP	NT	DS
Manufacturer	DETAX GmbH & Co. KG (Germany)	Nolatech (Russia)	Harz Labs (Russia)
3D printer	NextDent 5100; (3DSystem, USA)		
Software for constructing digital models	3DSprint (3DSystem, USA)		
3D printing technology	Digital Light Processing (DLP)		
Thickness of a single printed layer	50 µm		
Post-build processing	Anycubic Wash & Cure 2.0 (Cleaning with 70% isopropyl alcohol for 3 min, UV curing)		

**Table 2:** The mechanical properties of the 3D printed PMMA of the studied grades.

No.	Material	Flexural Modulus, GPa	Flexural Strength, MPa	Flexural Strain, %
1	FP	2.7 ± 0.6	90.7 ± 5.9	2.7 ± 0.6
2	NT	2.8 ± 0.1	41.7 ± 4.5	1.5 ± 0.1
3	DS	2.6 ± 0.3	104.2 ± 2.7	5.4 ± 1.2

**Table 3:** The adhesion indices of the normal, periodontopathogenic, and fungal microbiota to the materials under study.

No.	Material	Normal	Periodontopathogenic	Fungal
1	FP	0.55 ± 0.06	0.34 ± 0.05	0.43 ± 0.02
2	NT	0.56 ± 0.06	0.42 ± 0.05	0.49 ± 0.05
3	DS	0.43 ± 0.06	0.34 ± 0.05	0.34 ± 0.05

**Table 4:** The tribological properties of the PMMA dental materials in the point tribological contact.

No.	Material	Coefficient of Friction, CoF	Wear Rate, WR, mm^3^/N·m, 10^–5^	Wear Track Roughness, Ra, µm
1	FP	0.276 ± 0.019	13.52 ± 1.01	0.191 ± 0.030
2	NT	0.303 ± 0.025	26.97 ± 0.91	0.204 ± 0.026
3	DS	0.271 ± 0.022	28.29 ± 0.98	0.179 ± 0.015

**Table 5:** The tribological properties of the PMMA dental materials in the linear tribological contact.

No.	Material	Coefficient of Friction CoF	Wear Rate, WR, mm^3^/N·m, 10^–6^	Temperature, °C
1	FP	0.131 ± 0.018	0.120 ± 0.007	31.43 ± 1.50
2	NT	0.096 ± 0.016	0.078 ± 0.013	33.31 ± 0.21
3	DS	0.122 ± 0.018	0.176 ± 0.017	36.59 ± 0.99

**Table 6:** The summarized data on the WR values in the point, linear, and flat tribological contacts.

No.	Material	WR, Point Contact, mm^3^/N·m, 10^–5^	WR, Linear Contact, mm^3^/N·m, 10^–6^	(Abrasive) Weight Loss, Flat Contact, Δ*m*, gr
1	FP	13.52 ± 1.01	0.120 ± 0.007	0.121 ± 0.01
2	NT	26.97 ± 0.91	0.078 ± 0.013	0.255 ± 0.01
3	DS	28.29 ± 0.98	0.176 ± 0.017	0.193 ± 0.01

**Table 7:** The technological properties of 3D-printed PMMA materials.

No.	Material	Average Duration of 3D Printing and Post-Polymerization Processing, min	Roughness after Standard Polishing, Ra, µm	Warpage after 3D Printing (Quality)
1	FP	33 + 30 = 63	0.048 ± 0.005	–(0)
2	NT	33 + 50 = 88	0.049 ± 0.007	+(1)
3	DS	80 + 30 = 110	0.051 ± 0.003	–(0)

**Table 8:** The price for the dental material feedstocks (February 2023).

No.	Material	Price for 1 kg, US Dollar
1	FP	USD 381
2	NT	USD 203
3	DS	USD 404

**Table 9:** The alternatives, their factors, and the criteria for the initial data analysis.

Group	Factor	Criterion	Alternative		
			*A_1_* FP	*A_2_* NT	*A_3_* DS
Mechanical	Flexural modulus, GPa	1	2.7 ± 0.6	2.8 ± 0.1	2.6 ± 0.3
	Flexural strength, MPa	1	90.7 ± 5.9	41.7 ± 4.5	104.2 ± 2.7
	Flexural strain, %	1	2.7 ± 0.6	1.5 ± 0.1	5.4 ± 1.2
Tribological	Wear rate, point contact, WR, mm^3^/N·m, 10^–5^	–1	13.52 ± 1.01	26.97 ± 0.91	28.29 ± 0.98
	Wear rate, linear contact, WR, mm^3^/N·m, 10^–6^	–1	0.120 ± 0.007	0.078 ± 0.013	0.176 ± 0.017
	(Abrasive) weight loss, flat contact, Dm, gr	–1	0.121 ± 0.01	0.255 ± 0.01	0.193 ± 0.01
Technological	Average duration of 3D printing and post-build polymerization processing, min.	–1	63	88	110
	Roughness after standard polishing, Ra, µm	–1	0.05 ± 0.00	0.05 ± 0.00	0.05 ± 0.00
	Warpage after 3D printing (quality)	–1	0	1	0
Biological	Normal, c.u.	–1	0.55 ± 0.06	0.56 ± 0.06	0.43 ± 0.06
	Periodontopathogenic, c.u.	–1	0.34 ± 0.05	0.42 ± 0.05	0.34 ± 0.05
	Fungal, c.u.	–1	0.43 ± 0.02	0.49 ± 0.05	0.34 ± 0.05
Economic	Price for 1 kg of feedstock, USD.	–1	USD 381	USD 203	USD 404

**Table 10:** The data on the paired comparison and the weights. Mechanical factors.

Criterion	Flexural Modulus	Flexural Strength	Flexural Strain	Weight
Flexural modulus	1.00	1.50	1.50	0.43
Flexural strength	0.67	1.00	1.00	0.29
Flexural strain	0.67	1.00	1.00	0.29

**Table 11:** The data on the paired comparison and the weights. Tribological factors.

Criterion	Wear Rate, Point Contact	Wear Rate, Linear Contact	(Abrasive) Weight Loss, Flat Contact	Weight
Wear rate, point contact	1	1	1	0.33
Wear rate, linear contact	1	1	1	0.33
(Abrasive) weight loss, flat contact	1	1	1	0.33

**Table 12:** The data on the paired comparison and the weights. Technological factors.

Criterion	Average Duration of 3D Printing and Post-Polymerization Processing	Warpage after 3D Printing (Quality)	Weight
Average duration of 3D printing and post-build polymerization processing	1	0.33	0.25
Warpage after 3D printing (quality)	3	1	0.75

**Table 13:** The data on the paired comparison and the weights. Biological factors.

Criterion (Microbiota)	Normal	Periodontopathogenic	Fungal	Weight
Normal	1	0.11	0.20	0.06
Periodontopathogenic	9	1	5	0.72
Fungal	5	0.20	1	0.22

**Table 16:** The ranks according to the criteria of all groups. Preference #1/Preference #2/Preference #3.

No.	Alternative	S	R	Q(*v* = 0.5)	Rank	
					VIKOR	extVIKOR
*A*_1_	FP	[0.2294, 0.5132]/[0.3211, 0.5479]/[0.1966, 0.4728]	[0.0596, 0.1285]/[0.1803, 0.1803]/[0.0512, 0.0904]	[0.0000, 0.7666]/[0.4074, 0.7323]/[0.0000, 0.4566]	1/1/1	1/2/1
*A*_2_	NT	[0.5811, 0.7617]/[0.4964, 0.6702]/[0.6065, 0.8172]	[0.0829, 0.0907]/[0.0782, 0.1129]/[0.0958, 0.1348]	[0.4996, 0.7258]/[0.2510, 0.6381]/[0.5970, 1.0000]	1/2/2	1/1/2
*A*_3_	DS	[0.3591, 0.6200]/[0.4331, 0.6607]/[0.3097, 0.5864]	[0.0788, 0.1071]/[0.2035, 0.2035]/[0.0781, 0.0833]	[0.2613, 0.7115]/[0.6604, 0.9863]/[0.2519, 0.5060]	2/3/1	1/3/1

## Data Availability

The data presented in this study are available on request from the corresponding author.

## Supplementary Materials

The following supporting information can be downloaded at: https://www.mdpi.com/article/10.3390/ijms24076432/s1.
