# Supplementary material for: Dental Material Selection for the Additive Manufacturing of Removable Complete Dentures (RCD)
Source: Int J Mol Sci. 2023 Mar 29;24(7):6432. doi: 10.3390/ijms24076432 (PMC10094705; doi:10.3390/ijms24076432)
Supplement: Supplementary file 1 [file ijms-24-06432-s001.zip › ijms-2283801-supplementary.pdf]

International Journal of
Molecular Sciences

MDPI

Article

Dental Materials Selection for Additive Manufacturing of Polymer Removable Complete Dentures

Supplementary materials

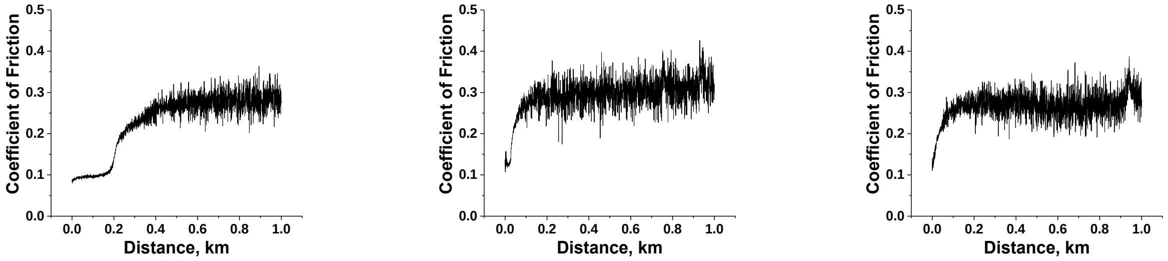

(**a**) (**b**) (**c**)

Figure S1. Time dependences of the CoF values for FP (**a**), NT (**b**), DS (**c**); the point tribological contact.

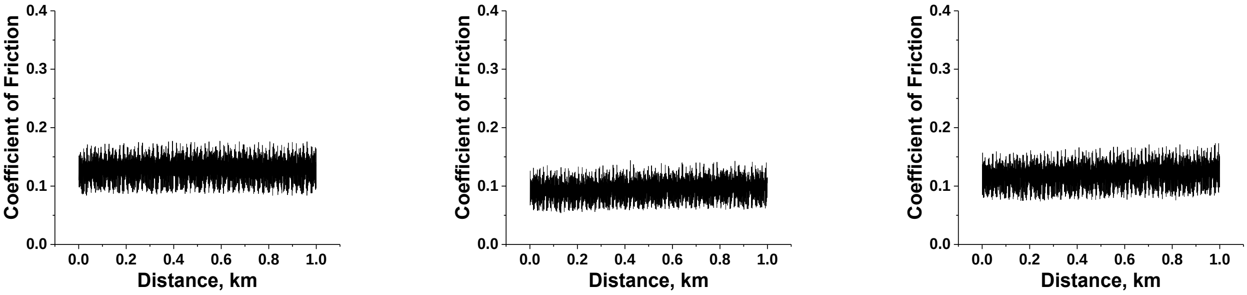

(**a**) (**b**) (**c**)

Figure S2. Time dependences of the CoF values for FP (**a**), NT (**b**), DS (**c**); the linear tribological contact.

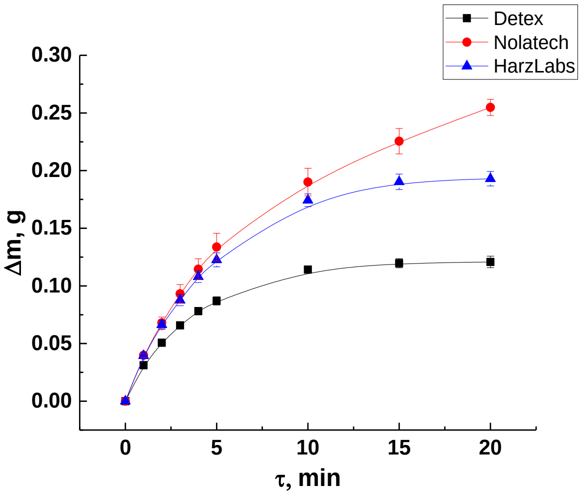

Figure S3. Time dependences of the PMMA (abrasive) weight loss. The flat tribological contact.

Table S1. The material indices calculated as the ratio of the mechanical properties to the feedstock cost.

Material	Flexural modulus	Flexural strength	Flexural strain
FP	0.7	23.8	0.7
NT	1.3	20.5	0.7
DS	0.6	25.7	1.3

Table S2. The material indices calculated as the ratio of the biological properties to the feedstock cost.

Material	Normal	Periodontopathogenic	Fungal
FP	0.2	0.1	0.1
NT	0.3	0.2	0.25
DS	0.1	0.1	0.08

Table S3. The material indices calculated as the ratio of the tribological properties to the feedstock cost.

Material	Wear rate, point contact	Wear rate, linear contact	(Abrasive) weight loss, flat contact
FP	3.5	0.3	0.031
NT	13.3	0.4	0.12
DS	6.9	0.4	0.047

Table S4. The material indices calculated as the ratio of the technological properties to the feedstock cost.

Material	Average duration of 3D printing and post-build polymerization processing	Roughness after standard polishing	Shape distortion after 3D printing (quality)
FP	16.5	0.126	0.26
NT	43.3	0.241	0.98
DS	27.2	0.126	0.25

Table S5. The ranks obtained by the extended VIKOR method according to the criteria within the groups. The estimated *S*, *R* and *Q* values within the groups.

No.	Alternative	S	R	Q (v=0.5)	Rank
Mechanical					
A_1	FP	[0.2235, 0.7664]	[0.1813, 0.4286]	[0.1301, 0.9850]	2
A_2	NT	[0.6664, 0.7857]	[0.2747, 0.2857]	[0.6380, 0.7500]	2
A_3	DS	[0.1429, 0.5111]	[0.1429, 0.3571]	[0.0000, 0.6614]	1
Tribological					
A_1	FP	[0.1250, 0.2449]	[0.1250, 0.1615]	[0.0000, 0.1684]	1
A_2	NT	[0.5595, 0.7067]	[0.2900, 0.3333]	[0.6894, 0.8926]	2
A_3	DS	[0.6950, 0.8658]	[0.2944, 0.3333]	[0.7912, 1.0000]	3
Technological					
A_1	FP	0.0000	0.0000	0.0000	1
A_2	NT	0.8830	0.7500	1.0000	3
A_3	DS	0.2500	0.2500	0.3082	2
Biological					
A_1	FP	[0.1329, 0.5985]	[0.1035, 0.4017]	[0.1381, 0.5770]	1
A_2	NT	[0.4826, 1.0000]	[0.3214, 0.7231]	[0.4635, 1.0000]	2
A_3	DS	[0.0000, 0.5174]	[0.0000, 0.4017]	[0.0000, 0.5365]	1
Economical					

A_1	FP	0.89	2
A_2	NT	0.00	1
A_3	DS	1.00	2
